# Meeting Report: Towards a Critical Assessment of Functional Annotation Experiment (CAFAE) for bacterial genome annotation

**DOI:** 10.4056/sigs.1323436

**Published:** 2010-11-29

**Authors:** Owen White, Nikos Kyrpides

**Affiliations:** 1 Institute for Genome Sciences, University of Maryland School of Medicine, Baltimore, MD 21201, USA; 2 DOE Joint Genome Institute, Walnut Creek, CA 94598, USA

## Abstract

It is widely recognized that, with the advent of very high throughput, short read, and highly parallelized sequencing technologies, the generation of new DNA sequences from microbes, plants, metagenomes is outpacing the ability to assign functions to (“annotate”) all this data. To begin to try to address this, on May 18 and 19, 2010, a team of roughly fifty people met to define and scope the possibility of a first Critical Assessment of Functional Annotation Experiment (CAFAE) for bacterial genome annotation in Crystal City, Virginia. Due to the fundamental importance of genomic data to its mission, the Department of Energy (DOE) BER program hosted this workshop, funding the attendance of all invitees. The workshop was co-organized by Dan Drell and Susan Gregurick (DOE), Owen White and Nikos Kyripides.

## Goal of the meeting

The CAFAE meeting explored the utility of a competition process, somewhat modeled on the Critical Assessment of Techniques for Protein Structural Prediction (CASP) [1], to advance the science of gene and genome annotation. The goal of this meeting was to plan an international effort to review and assess the quality of bacterial annotation, to improve current annotation practices by rigorous comparison of the diverse set of methods that have been developed by the genome community, and to explore how a competition structure might serve to accelerate improvements in bacterial genome annotation. To this end, the participants sought to form a general framework, led by an international committee to structure and supervise a potential gene/genome annotation competition; and to establish rules of engagement and a time-line for participation in a CAFAE competition.

## Participants

The meeting was attended by a diverse set of individuals representing many disciplines including genome annotation, large-scale DNA sequence generation, describing ontologies and defining genome standards, experienced bench scientists, representatives from several public DNA and protein sequences archives, as well as a large complement of funding agency representatives, and staff from the White House Office of Science and Technology Policy (OSTP). Participants were asked to prepare one-page reports and short Powerpoint talks in preparation for the meeting. The format of the meeting included plenary presentations, breakout sessions, as well as ad hoc sessions for brainstorming and consensus building.

## Meeting outcomes

Several conclusions were achieved at the meeting. Virtually all attendees agreed that there is a significant need for the CAFAE competition and it was quite likely that many members of the annotation community would participate. Several tactical decisions were made including methods to enable exchange of genes, annotations and the experimental and computational evidence that are used to establish annotations. The group was also confident that domain experts could be recruited to participate in accuracy measurement/assessments of CAFAE annotations, as well as to collect critical sources of experimentally validated genes that could serve as the basis of reviewing *in silico* gene annotations from the CAFAE participants. The resources and willingness to perform data management required to evaluate the annotation data were also identified.

**Key steps towards building the CAFAE community and its infrastructure must include:**
1. Formation of a mechanism of governance. This would involve the selection of a Steering Committee to oversee project management, governance, resource gathering, outreach, and recruitment for participation in the competition. The Steering Committee must be diverse and international.
2. Creation of a defined competition. To do this CAFAE organizers must gather annotations from a number of contributors from the user community. The data received from the submitters must be evaluated by a number of relevant metrics, which will include tests for accuracy, confidence, completeness, and consistency. Methods to validate these metrics will be devised in the context of experimentally validated data sets that are contributed from the larger biological community, and the experimentally validated datasets will be integrated into a richer set of annotations that will be made available to the broad scientific community.

While recent developments in genome technologies applied to microbes are generating enormous amounts of sequence data, there are many cases of individual laboratories that are also generating experimental information. The distributed nature of these data sources creates difficulties in data sharing, despite the best of intentions, such that much time is likely to be spent trying to determine the experimentally derived data that is associated with genes from whole genomes, as well as retrieving the experimental information that is associated with closely related species of bacteria where genes are likely to have identical functions. Given that the rate of data production is rapidly outpacing analysis, this trend is sure to become worse.

In addition to the enthusiasm for the launching of CAFAE, perhaps the most exciting development of the meeting was the recognition by all of the attendees that CAFAE could easily serve as a broker for accepting large-scale computational annotation associated with bacterial sequences, and at the same time associate this information, in a defined and transparent manner, with experimentally derived data. This would be achieved by the following steps:

1. Enabling genome annotation producers to submit their data in a common repository using rigorous data submission tools that describe the evidence that is used to support any annotation.
2. Enabling the research community to use nearly identical data submission methods to submit their experimental results and linking the experimental results with annotations.
3. Evaluating the quality of the annotation with a relatively simple set of procedures that assess the quality of the *in silico* predictions, but also by using the presence of any experimental data to review the accuracy of those annotations.
4. All experimental data, annotations, improved/refined annotations would then be available in a single common infrastructure that would be accessible to the user community.
5. All improved/refined annotations which have been validated against experimentally verified genes would also be available to the annotation community, so annotation producers could use these data in subsequent rounds of processing.

Probably the single most important realization for the CAFAE meeting participants was that this project could rapidly link large sets of experimentally characterized genes to annotations, and to maximize this information to refine our annotation methods while at the same time creating a CAFAE clearinghouse that would be extremely useful to the biological community.

Any future CAFAE competition and the concomitant infrastructure will be specifically designed to receive the experimentally validated gene information from both the suppliers of the gene validations as well as from the annotation predictors. In addition to this, the infrastructure will drive identification of functions for unknown sets of genes, because the CAFAE repository will present a large set of predictions that are poised for experimental validation. In short, unvalidated genes with annotations predicted as “high priority” can become the focus of intensive experimental study to accelerate their characterization, thus serving various agency science missions. We also expect that by closely coupling experimental validation to the annotation process, many other new potential synergies will emerge, including a connection with the developing Systems Biology Knowledgebase [2] as well as the GenBank repository, through third party annotations. The meeting attendees also insisted on a general governance structure that could deal with issues of fairness, impartiality, outreach, publications and competition rules.

The CAFAE meeting included representatives from non-government organizations – the Gordon and Betty Moore Foundation and the X Prize Foundation. Grant Campany from the X Prize Foundation recommended that CAFAE consider the X Prize model as part of its future plans. This model identifies a clearly defined and delineated goal and then establishes a monetary award for its accomplishment. He also noted that there is keen interest, from the government perspective, in prize development to address key problems in society, research and government. He also stated that the CAFAE competition component was quite likely to serve as the basis of establishing milestones amenable to X Prize competitions, and he encouraged forward-thinking to create a vision for technologies that could be achieved within five years with an X Prize mechanism as an incentive to achieve that vision.

If this community can be mobilized to put in place the agreements and infrastructure required to support repeated CAFAE competitions, the outcomes could include a step-change improvement in the transparency, quality and quantity of genomic annotations. It would also forge new links between the experimental and genomic annotation communities as well as eventually produce, through combined annotations taken from ‘best of breed’ methodologies, some of the richest and most comprehensive, community-vetted genome annotations ever produced.

## References

[1] Kryshtafovych A Krysko O Daniluk P Dmytriv Z Fidelis K Protein structure prediction center in CASP8. Proteins 2009; 77(Suppl 9):5-9 19722263 10.1002/prot.22517 PMC2863353

[2] Systems Biology Knowledgebase DOE (http://genomicscience.energy.gov/compbio/).

